# First Dimension Trap‐and‐Elute Combined with Second Dimension Reversed‐Phase Liquid Chromatography Separation Using a Two‐Dimensional‐Liquid Chromatography‐Tandem Mass Spectrometry System for Sensitive Quantification of Human Insulin and Six Insulin Analogs in Plasma: Improved Chromatographic Resolution and Stability Testing

**DOI:** 10.1002/jssc.70092

**Published:** 2025-02-10

**Authors:** Pavel Sistik, Romana Urinovska, Klara Handlosova, Petr Handlos, Katerina Andelova, Jan Jurica, David Stejskal

**Affiliations:** ^1^ Department of Clinical Pharmacology Institute of Laboratory Medicine University Hospital Ostrava Ostrava Czech Republic; ^2^ Institute of Laboratory Medicine Faculty of Medicine University of Ostrava Ostrava Czech Republic; ^3^ Department of Clinical Pharmacology Faculty of Medicine University of Ostrava Ostrava Czech Republic; ^4^ Department of Forensic Medicine University Hospital Ostrava Ostrava Czech Republic; ^5^ Department of Forensic Medicine Faculty of Medicine University of Ostrava Ostrava Czech Republic; ^6^ Institute of Laboratory Medicine University Hospital Ostrava Ostrava Czech Republic; ^7^ Department of Pharmacology Faculty of Medicine and Department of Pharmacology and Toxicology Faculty of Pharmacy Masaryk University Brno Czech Republic

**Keywords:** human plasma, insulins, liquid chromatography, mass spectrometry, method validation, stability studies

## Abstract

Multidimensional chromatography coupled to tandem mass spectrometry (MS/MS), including simple sample preparation with protein precipitation, anion conversion with ammonium hydroxide, and solid‐phase extraction using mixed‐mode anion exchange in a 96‐well plate format, has been validated for rapid simultaneous analysis of human insulin and its six analogs (lispro, glulisine, glargine, degludec, detemir, and aspart) in human plasma. This method is critical for clinical diagnostics, forensic investigations, and anti‐doping efforts due to the widespread use of these substances. In the present study, improved chromatographic resolution was achieved using a first‐dimension trap‐and‐elute configuration with an XBridge C18 (2.1 × 20 mm, 3.5 µm) trap column combined with second dimension separation on a Cortecs Ultra‐High‐Performance Liquid Chromatography (UHPLC) C18+ (2.1 × 100 mm, 1.6 µm) analytical column implemented within a two‐dimensional‐LC‐MS/MS system. The total chromatographic run time was 11 min. This setup increases both the resolution and sensitivity of the method. A mobile phase consisting of 0.8% formic acid (FA) in water and 0.7% FA in acetonitrile was used for gradient elution. Bovine insulin was used as the internal standard. MS detection was performed in positive electrospray ionization mode, and the ion suppression due to matrix effects was evaluated. Validation criteria included linearity, precision, accuracy, recovery, lower limit of quantitation, matrix effect, and stability tests with and without protease inhibitor cocktail under different conditions (short‐term stability, long‐term stability, and freeze‐thaw stability). The concentration range for all insulins was 50–15 000 pg/mL, with limits of quantification below the therapeutic reference range for all analytes. Intra‐run precision ranged from 1.1% to 5.7%, inter‐run precision from 0.7% to 5.9%, and overall recovery from 96.9% to 114.3%. The validated method has been implemented successfully by the Department of Forensic Medicine at our hospital for the investigation of unexplained deaths.

**Abbreviations**
µSPE: micro solid‐phase extraction
2D‐LC‐MS/MS: two‐dimensional liquid chromatography‐tandem mass spectrometry
BSA: bovine serum albumin
CE: collision energy
CV: coefficient of variation
EMA: European Medicines Agency
ESI: electrospray ionization
FA: formic acid
HQC: high quality control
HRMS: high‐resolution mass spectrometry
IDE: insulin‐degrading enzyme
UPLC: ultra‐performance liquid chromatography
LLOQ: lower limit of quantitation
LQC: low quality control
MAX: mixed‐mode anion exchange
MRM: multiple reaction monitoring
PIC: protease inhibitor cocktail

## 1 Introduction

Insulin, a peptide hormone produced by the pancreas, plays a crucial role in regulating blood glucose levels by facilitating the uptake of glucose into cells for energy. Insufficient insulin production or resistance to its effects results in diabetes mellitus, a chronic metabolic disorder characterized by high blood glucose levels [1]. Insulin delivery has grown increasingly important in recent years due to the increasing global prevalence of diabetes mellitus. This escalating burden underscores the need for effective insulin therapy and continued research to improve diabetes management [2]. Insulin analogs are synthetic derivatives designed to replicate the action profile of natural insulin while offering improved pharmacokinetic properties. The chemical structures of the analyzed insulin analogs are provided in Figures S1–S7 to illustrate their molecular differences and structural similarities. Various insulin options are available for different species, including veterinary‐approved products [3].

Under doping control regulations, insulin is considered a prohibited substance in both animal sports (such as horse and dog racing) and professional sports (such as athletics [4] or cycling) due to its potential to enhance muscle growth and athletic performance. As a result, insulin is routinely monitored in equine plasma and urine [5, 6].

Anti‐doping authorities use a variety of analytical techniques, including urine and blood tests, to detect insulin abuse in athletes. These methods aim to measure insulin levels and identify the presence of insulin analogs [7].

Insulin has also attracted attention in forensic medicine in cases of unexplained death. Accidental overdoses are often due to patient error, whereas intentional overdoses, particularly in suicide attempts, may be underestimated in individuals with diabetes. Research suggests that insulin is often used as a method of self‐poisoning, particularly in individuals with type 1 diabetes [8].

Analysis of insulin in blood by immunoassays is common practice in routine clinical laboratories. However, these tests should be interpreted with caution, especially in patients taking insulin analogs. The main problem is cross‐reactivity with other substances in the blood, which can lead to inaccurate results. Another factor is hemolysis, the breakdown of red blood cells, which can distort the measurement [9]. Rosil compared seven commercially available insulin immunoassays and highlighted significant differences in their results. The authors emphasized the need to standardize methods of analyzing insulin and improve their mutual comparability [10].

Several liquid chromatography‐mass spectrometry (LC‐MS) methods have been developed to detect insulins and their analogs in human plasma [11, 12], serum [13], urine [14], and vitreous humor [15, 16, 17]. Targeted insulins are typically isolated from plasma or urine by solid‐phase extraction (SPE) and/or immunoaffinity purification techniques. Subsequently, LC‐MS is employed to analyze multiply charged intact insulins and their B‐chains. A number of proteomic methods for high‐resolution MS (HRMS) analysis of insulins have been documented [11, 18]. However, from a clinical laboratory perspective, these methods can be time‐consuming due to lengthy sample preparation procedures or multi‐step workflows.

The volume of the biological matrix used for the determination of insulin ranges from 250 to 500 µL for plasma [12, 19], and the matrix volume varies in units of milliliters for urine [14]. In the clinical laboratory, multi‐method quantification analysis (analyzing several substances in one analysis) [12, 17, 19, 20] has priority over single‐method analysis [21].

Given the very similar structures of human insulin and its recombinant analogs and their fragmentation spectra in MS, effective column separation is desirable. Few studies have successfully separated the two critical insulins (human and lispro) [14], and many published papers have not achieved column separation [12, 19]. Therefore, they had to use specific fragments for quantification. The stability of insulin and its structural analogs in biological matrices, particularly human fluids, is a well‐known problem and is often attributed to enzymatic degradation by enzymes, such as an insulin‐degrading enzyme (IDE) and peroxisomal protease [22, 23]. Comprehensive stability testing under different conditions is rarely addressed in the literature, despite its importance [24, 25].

The aim of the present study was to develop and validate a rapid, simple, and sensitive method for the determination of human insulin and its six structural analogs using a first‐dimension trap‐and‐elute configuration combined with second‐dimension reversed‐phase LC (RPLC) separation within a two‐dimensional LC‐tandem MS (2D‐LC‐MS/MS) system. We attempted to separate these substances, increasing the sensitivity by using fragments with a higher response in MS/MS, thereby increasing the overall sensitivity of the determination. The method development was based on the requirements of the Department of Forensic Medicine in cases of unexplained deaths. In addition, we evaluated the plasma stability of human insulin and six analogs under different experimental conditions, both with and without protease inhibitor cocktail (PIC). This research should provide valuable information for optimizing insulin quantification and increasing the reliability of experimental results.

## 2 Materials and Methods

### 2.1 Chemicals and Reagents

LC‐MS water for the mobile phase and sample preparation was purchased from Honeywell Chemicals (Seelze, Germany). Acetonitrile, methanol, formic acid (FA) (≥99%), and acetic acid (≥99%) were purchased from VWR (Prague, Czech Republic). LC‐MS grade ammonium hydroxide solution (25%), bovine serum albumin (BSA), and PIC were purchased from Merck (Prague, Czech Republic). Artificial plasma (BZ273) for method validation was obtained from Biochemazone (Canada). Human insulin, bovine insulin, insulin aspart, insulin lispro, and insulin glargine were obtained from Merck (Prague, Czech Republic). Commercially available glulisine (Apidra, Sanofi‐Aventis Deutschland GmbH) as a 3.49 mg/mL solution, detemir (Levemir, Novo Nordisk A/S, Novo Allé, DK‐2880 Bagsværd, Denmark) as a 14.2 mg/mL solution, and degludec (Tresiba, Novo Nordisk A/S, Novo Allé, DK‐2880 Bagsværd, Denmark) as a 7.32 mg/mL solution were obtained from the pharmacy of the University Hospital Ostrava, Czech Republic. An Oasis mixed‐mode anion exchange (MAX) 96‐well µElution Plate, 2 mg sorbent per well, 30 µm (Waters, Prague, Czech Republic) was used for sample preparation. QuanRecovery with MaxPeak 700 µL plates (Waters, Prague, Czech Republic) was used after the extraction, and the samples were injected from these plates. All insulin stock solutions were prepared and stored in 2.0 mL Protein LoBind tubes (Eppendorf, Czech Republic).

### 2.2 Multidimensional LC and MS Detection

Multidimensional chromatography was performed using two binary ACQUITY UPLC I‐Class Plus systems (Waters) configured in a first‐dimension trap‐and‐elute setup with an XBridge C18 (2.1 × 20 mm, 3.5 µm) trap column (Waters) followed by second dimension separation on a Cortecs UPLC C18+ (2.1 × 100 mm, 1.6 µm) analytical column (Waters). The ACQUITY UPLC 2D system with 2D valve technology was used for trap‐and‐back‐elute chromatography. During the loading phase (0–2 min), the sample was introduced onto the trapping column in loading mode (Figure S8). The valve was then switched to eluting mode (2–8.5 min) (Figure S9), where the analytes were back‐eluted onto the analytical column. After 8.5 min, the valve was returned to the loading position.

The gradient elution solvents consisted of 0.8% FA in water (mobile phase A) and 0.7% FA in acetonitrile (mobile phase B). The trapping solvent consisted of 85:15 mobile phase A:B at a flow rate of 0.1 mL/min. A 30 µL sample was injected into the trapping column for 2 min, followed by switching the valve to position 2 for back eluting onto the analytical column maintained at 60°C, with an initial isocratic elution using 25% B over 4 min at 0.3 mL/min. This was followed by a gradient increase from 25% to 50% B between 4.1 and 5.5 min. During the next 0.5 min, the gradient was further increased to 95% B and held for 1.5 min for cleaning. Finally, the system was re‐equilibrated at baseline conditions for 1.5 min. The autosampler was set at 6°C.

The ultra‐performance liquid chromatography (UPLC) systems were connected to a Xevo TQ‐XS triple quadrupole mass spectrometer (Micromass, Manchester, UK). The analytes in the eluate from the UPLC column were introduced into a capillary sprayer using positive electrospray ionization (ESI+). The source and desolvation temperatures were set to 150°C and 600°C, respectively. The capillary voltage was maintained at 1.5 kV, whereas the cone voltage ranged from 30 to 60 V and the collision energy (CE) varied from 18 to 50 eV. The cone voltage and CE values for the qualification and quantification fragments of all insulins are shown in Table 1. Nitrogen was used at a flow rate of 0.15 mL/min and multiple reaction monitoring mode. The MS data were processed using MassLynx V4.2.

**TABLE 1:** Insulins and their multiple reaction monitoring (MRM) transitions together with cone voltage (CV) and collision energy (CE) settings, highlighted transitions are quantification transitions.

Specific insulin	MRM transition	Cone Voltage (V)	Collision energy (eV)
Glargine	867.00 => 136.20	60.00	40.00
	**1011.30 => 1179.00**	60.00	25.00
Lispro	**968.80 => 136.1**	60.00	40.00
	1162.3 => 217.20	60.00	40.00
Human insulin	968.90 => 136.10	30.00	50.00
	**1162.40 => 226.00**	50.00	40.00
Aspart	972.00 => 136.00	30.00	50.00
	**972.00 => 661.20**	30.00	18.00
Glulisine	**1165.50 => 346.20**	30.00	26.00
	1165.50 => 1370.10	30.00	20.00
Detemir	1184.40 => 454.40	50.00	30.00
	**1184.40 => 1366.70**	50.00	20.00
Degludec	1221.80 => 641.40	30.00	32.00
	**1221.80 => 1366.60**	30.00	24.00
Bovine (IS)	**956.50 => 1121.00**	60.00	18.00

### 2.3 Preparation of Stock Solutions, Calibration Standards, and Quality Control Samples for Method Validation and Stability Testing

Individual stock solutions of insulins and the internal standard (bovine insulin, BSA) were prepared in a mixture of water, acetonitrile, and FA (59.98:40:0.02, v/v/v) with 5 µg/mL BSA and stored at −80°C. The concentrations of the stock solutions of all insulins were 1 mg/mL. The internal standard (bovine insulin) working solution was prepared in the same diluent at a concentration of 100 ng/mL for spiking and stored at −80°C.

A mixed standard solution containing all insulins at a concentration of 10,000 ng/mL was obtained by diluting the stock solutions with artificial plasma. This stock solution was further diluted with artificial plasma to produce a series of calibration standards at the following concentrations: 50, 100, 150, 250, 500, 1,000, 1500, 2500, 5000, 10 000, and 15 000 pg/mL. Quality control (QC) samples were prepared in artificial plasma at 200, 800, 3000, and 9000 pg/mL and analyzed prior to real human plasma samples. For method validation, four concentration levels were prepared in artificial plasma at 50, 125, 6000, and 13 000 pg/mL. Two concentration levels (193 and 7752 pg/mL) were evaluated for stability testing. One set of tests was performed with artificial plasma and the other set with artificial plasma containing 1% PIC.

### 2.4 Sample Preparation

Patient samples were collected in K_2_EDTA tubes, and centrifuged, and 250 µL aliquots were either analyzed immediately or stored at −80°C for analysis the following day. A previously published method [12] was used for sample preparation, including protein precipitation, conversion of analytes to anions, and micro SPE (µSPE) using Oasis MAX multimodal sorbent (Waters) in a 96‐well design. The MAX sorbent is a mixed‐mode material that combines reversed‐phase retention with anion exchange properties. This dual mechanism allows efficient extraction of acidic analytes from complex biological matrices, providing high selectivity and recovery. The polymeric structure provides chemical stability, making it suitable for a variety of sample preparation needs. QuanRecovery with MaxPeak 700 µL plates (Waters) were used for elution after SPE and subsequent injection for 2D‐LC‐MS/MS analysis. These plates have a special coating that minimizes protein binding, ensuring higher analyte recovery and reducing sample loss during preparation.

To assess the clinical applicability of the proposed method, 30 patient samples (16 males and 15 females) were analyzed. Plasma samples (collected in K_2_EDTA tubes) were supplied to the Department of Forensic Medicine.

### 2.5 Method Validation

Method validation, including linearity, precision, accuracy, recovery, lower limit of quantitation (LLOQ), matrix effect, and different stability testing conditions with and without PIC, was performed according to the European Medicines Agency (EMA) guidelines for bioanalytical method validation [26]. Artificial plasma fluid (BZ273) was used for the method validation.

#### 2.5.1 Linearity and LLOQ

Calibration curves for all analytes were constructed using weighted least squares linear regression analysis (1/*x* weighting) by plotting the peak area ratios of the analyte to the internal standard against the nominal concentration of the calibration standards. The calibration curves consisted of 11 calibration points evenly distributed over the dynamic range.

The LLOQ is defined as the lowest concentration with acceptable precision and accuracy. For this study, the criteria for the LLOQ were a coefficient of variation (CV) <20% for precision and accuracy in the range of 80% to 120%.

#### 2.5.2 Accuracy and Precision

To evaluate accuracy and precision, four concentration levels were prepared in artificial plasma at 50, 125, 6000, and 13,000 pg/mL and used for method validation. Ten replicates of each level were analyzed on the same day to determine repeatability and intra‐assay precision and on three different days to assess inter‐assay precision and recovery. The precision (CV) of the method had to be within 15% and the accuracy had to be within 15% of the nominal concentration.

#### 2.5.3 Matrix Effect

To investigate potential ion suppression effects from the matrix, six batches of samples were tested as follows: Sample A consisted of a precipitation mixture containing water, and Sample B consisted of a precipitation mixture containing artificial plasma. All samples were spiked with 50 µL of an insulin mixture at two concentrations (lowest and highest) after µSPE. The same procedure was used to prepare samples to assess the matrix effect on the internal standard. The matrix factor was calculated for both the analytes and the internal standard, with the peak areas of the matrix‐free samples set at 100%. According to EMA guidelines, the matrix factor should not exceed ±15% for both concentrations.

#### 2.5.4 Stability

The stability tests of all insulins were performed at two concentration levels (193 and 7752 pg/mL). One set of tests was performed with artificial plasma and the other set with artificial plasma containing 1% PIC. Each concentration level was tested in triplicate. These QC samples were quantified using an artificial plasma calibration curve without PIC, and the obtained concentrations were compared with the nominal concentrations of the low quality control (LQC) and high quality control (HQC), which were set to 100%. To evaluate the stability of insulins in artificial plasma containing 1% PIC, we considered the concentration measured at time zero as the baseline (100%). All subsequent changes in concentration were calculated relative to this initial measurement.

Short‐term stability, long‐term stability, and freeze‐thaw stability were evaluated in this study. LQC and HQC samples were stored at room temperature and in a refrigerator for 3, 6, and 24 h to assess short‐term (bench‐top) stability and at 4°C in the autosampler for 24 h to assess re‐injection stability. To assess freeze‐thaw stability, samples stored at −80°C were subjected to three freeze‐thaw cycles (24, 48, and 72 h) and then analyzed. For long‐term stability (except insulin glargine), samples were stored at −20°C and −80°C for 1, 2, and 3 weeks.

## 3 Results and Discussion

### 3.1 Chromatographic and MS Conditions

Ionization parameters, cone voltages, and CEs were systematically optimized using post‐column infusion (0.2 mL/min) of the highest concentration solution of each standard in ESI+ mode. A flow rate of 0.2 mL/min of 50:50 mobile phase A:B was used. Under these conditions, 4‐ to 8‐times protonated molecules were obtained for the insulins. Quantification and qualification of the precursor and product ion masses, cone voltage, and CE for each substance are given in Table 1. The multiple reaction monitoring (MRM) mode was used for quantification.

**FIGURE 1:**
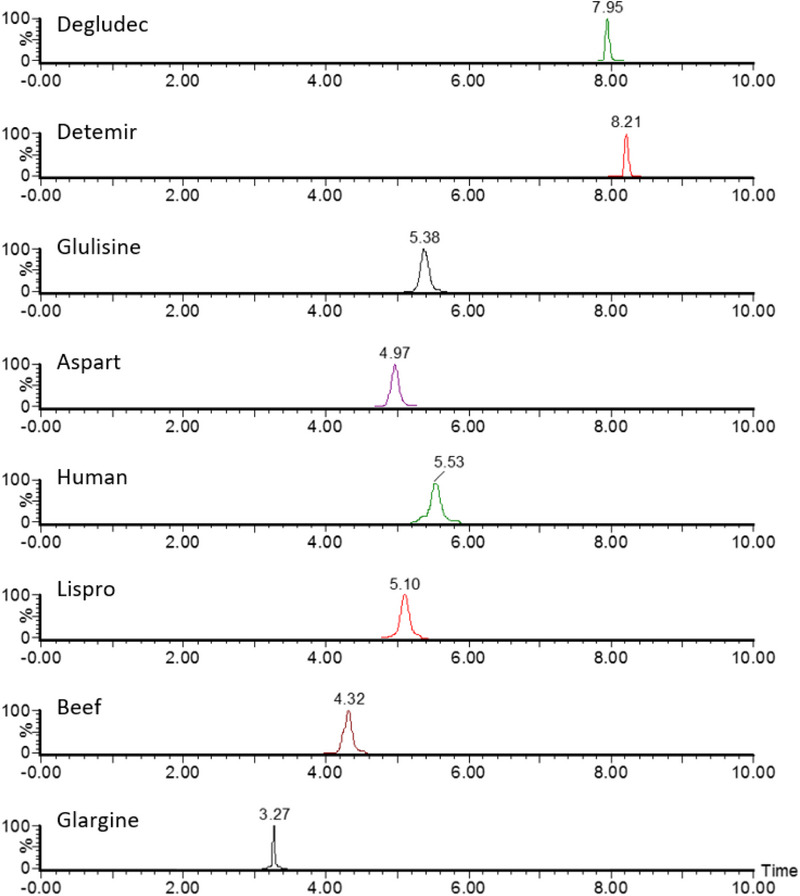
Chromatograms obtained using a two‐dimensional liquid chromatography‐tandem mass spectrometry (2D‐LC‐MS/MS) system with trap‐and‐elute configuration in the first dimension and reversed‐phase liquid chromatography (RPLC) separation in the second dimension for artificial plasma spiked with the highest concentrations of insulin standards (15 000 pg/mL).

During the development of the chromatographic method, six analytical columns and different mobile phase compositions and elution gradients were tested to achieve optimal multidimensional chromatographic separation and improve the peak symmetry for all insulins. Product ion chromatograms obtained for artificial plasma containing the highest concentration of standards (15 000 pg/mL) demonstrated sufficient separation and rapid analysis (Figure 1). In addition, a chromatogram representing the separation at the LLOQ concentration of 50 pg/mL for all analytes is provided in Figure S12 to illustrate the sensitivity and resolution of the method. The use of the trap‐and‐back‐elute configuration significantly improved the chromatographic separation of critical insulin pairs, such as human insulin and lispro. This approach is consistent with strategies reported in the literature [12] and demonstrates the utility of multidimensional systems for complex peptide analysis. During method development, this separation could not be achieved using 1D chromatography alone, even after extensive optimization of columns and gradients. Examples of the use of 1D chromatography for the separation of insulin lispro and human insulin are shown in Figure S11.

The human insulin molecule and its recombinant analogs are very similar in structure and, therefore, have similar physicochemical properties. For example, the peptide structures of human insulin and insulin lispro differ by only two amino acids. These insulins have identical masses and can only be distinguished by the presence of their specific MS^2^ diagnostic ions (y2 fragment ions). Most current identification methods rely solely on these unique fragment ions [12]. However, when analyzing these two insulins, alternative fragment ions are only viable for quantification if complete separation occurs on the LC column. For example, the tyrosine immonium ion (*m/z* 136) appears in the fragmentation spectra of not only these two substances but also others. Although it has a higher response in the MS spectrum, making it a more suitable fragment for quantification, tyrosine immonium can only be selected for determination by MS if effective column separation is achieved. Although successful cases of column separation have been reported [14], many authors have encountered difficulties, even with multi‐dimensional chromatography [12]. To overcome this problem, we sought to improve the separation of these insulins under multi‐dimensional chromatography conditions. After evaluating different columns and ultra‐high‐performance LC (UHPLC) gradients, optimal separation was achieved using an Acquity Cortex C18 column (100 × 2.1 mm, 1.7 mm) and refinement of the gradient originally proposed by Chambers et al. [12], resulting in a total chromatographic analysis time of 11 min.

To avoid false positive results due to the similarity of the MRM transitions of insulins, we tested the selectivity of optimized MRM transitions during method development. Initially, achieving separation of all analytes, especially the critical pair of lispro and human insulin, proved challenging. Selectivity testing was therefore essential to confirm the identification and quantification of specific analytes. However, after further optimization of the chromatographic conditions, complete column separation of all analytes was achieved. Adequate assay specificity was achieved through a combination of specific MRM transitions and chromatographic separation. While the MRM transitions provided initial selectivity, the chromatographic separation was critical to ensure complete resolution of the analytes, particularly for closely related pairs such as insulin lispro and human insulin. Based on this improved separation, we selected the more sensitive *m*/*z* 136 as the quantification product ion for insulin lispro, although this fragment was also observed for human insulin. The other MRM transitions of the remaining insulins were specific due to the effective column separation, eliminating the risk of co‐elution and ensuring accurate quantification.

### 3.2 Sample Preparation

Immunopurification [14, 20] and SPE [12] are common sample preparation techniques for the determination of insulin and its analogs from a biological matrix. These methods differ in selectivity, capacity, sensitivity, and other aspects. Immunopurification relies on the specific interaction between the antibody and the insulin target molecule to achieve high selectivity for specific analogs. The use of antibodies for extraction enhances the specificity of the methods considerably. This is very important for complex matrices but can be time‐consuming and expensive. However, low capacity and non‐specific binding of other proteins can lead to a loss of sensitivity and bias in the results. This method is suitable for the analysis of specific analogs but less suitable for the determination of total insulin in a complex matrix. In contrast, µSPE uses a sorbent with a high affinity for insulin to selectively capture it from the matrix. It typically offers higher capacity and lower non‐specific binding, improving sensitivity and reducing noise. This method is more universal and suitable for the determination of total insulin and its analogs. The MAX sorbent is commonly used in sample preparation for biological insulin samples [12, 17]. However, the author also used mixed‐mode cation exchange cartridges for LC‐HRMS analysis of human insulin and its synthetic analogs [19, 27].

Insulin and its analogs have a tendency to adsorb to surfaces, including plastic and glass tubes. This non‐specific adsorption can lead to loss of insulin and bias experimental results, especially at the low therapeutic concentrations found in plasma. Studies have shown that insulin adsorbs to surfaces, so it is important to use appropriate containers to mitigate this effect [28]. The use of Protein LoBind tubes and QuanRecovery vials or plates is recommended to suppress hydrophobic non‐specific binding. During the development of our method, we used low‐binding Eppendorf tubes and, after µSPE, we eluted the samples into QuanRecovery using MaxPeak 700 µL plates. We prepared stock solutions of all insulins, which were then transferred to three different types of glass vials and analyzed by LC‐MS/MS. For the adsorption test, a mixture of all insulins at a concentration of 15 ng/mL was prepared in a solvent containing 6.7% acetic acid in a water:methanol mixture (60:40, v/v). The mixture was then transferred to three types of vials and analyzed by UHPLC‐MS/MS. This experiment was performed prior to the optimization of the chromatographic method. Due to incomplete separation at this stage, human insulin and insulin lispro were prepared and tested individually in separate vials rather than in the mixture. The concentrations of some insulins differed by up to 50% (e.g. human insulin and lispro), indicating significant adsorption to the surfaces of the standard containers. This issue is often overlooked but is critical for the accurate quantification of insulin in biological samples. The results of this experiment are shown in Figure S10.

### 3.3 Method Validation

Validation of the proposed method, including linearity, precision, accuracy, recovery, LLOQ, matrix effect, and different stability test conditions (with and without PIC), was performed according to the EMA guidelines for validation of bioanalytical methods. For these purposes, we used an artificial plasma (BZ273) whose controlled composition mimics the basic properties of human plasma (pH, proteins, electrolytes). This reduces variability and facilitates standardized comparisons between experiments, which is supported by publications by other authors [29, 30, 31].

We also verified the specificity of the method on real plasma samples from healthy volunteers and insulin‐treated patients to confirm that there is no interference with the observed signals under clinical conditions. The results confirmed the robustness and applicability of the method in clinical practice, and the validation met the required criteria in the range of patient‐relevant concentrations.

Since human insulin is commonly present in real samples and can complicate stability testing, artificial plasma (BZ273) allowed us to better control conditions and obtain reliable data on the stability. We have also successfully used this surrogate matrix for other clinically relevant analytes (e.g., metanephrines and catecholamines) at our clinical laboratory in the hospital. At the same time, numerous studies [29, 30, 31] show that artificial plasmas provide consistent and reproducible conditions for the development of various bioanalytical methods.

While we recognize that artificial plasmas do not fully reproduce the complex composition of real plasma, their use is supported by the available literature. The publication by San Román et al. deals with the analysis of per‐ and polyfluoroalkyl compounds [29]. Natarajan et al. test biomarkers for brain injury [30] and Yang et al. evaluate sensors for therapeutic drugs [31]. These examples illustrate the practical benefits and broad applications of synthetic matrices.

Aware of the potential limitations, we chose artificial plasma primarily to avoid interference from endogenous human insulin and to determine the stability of insulin and its analogs. This approach is consistent with published evidence and regulatory recommendations and helps to achieve consistent and reproducible results. Regulatory guidelines, including those of the EMA [26] and Food and Drug Administration [32], support the use of surrogate matrices such as artificial plasma, provided they are validated and properly justified. These matrices ensure reliable and reproducible method performance in analytical applications. At the same time, we recognize that full validation also requires testing on real samples.

#### 3.3.1 Linearity and LLOQ

Linearity was evaluated for each analog over the concentration range of 50 to 15 000 pg/mL. The linearity of the calibration curves was statistically verified with correlation coefficients ≥0.998 for all insulins. Table 2 summarizes the correlation coefficient, intra‐day, and inter‐day precision and accuracy of the validation assay for each insulin. The first points of the calibration curves for all analytes met the criteria of accuracy within 80%–120% and precision with a CV < 20% and, therefore, were accepted as the LLOQ. The CVs for intra‐day and inter‐day precision for all insulins ranged from 0.7% to 5.9%, and the recovery values ranged from 96.9% to 114.3%.

**TABLE 2:** Parameters of validation (CV‐ coefficient of variation and R‐ recovery).

Analyte	Correlation coefficient *r*	Concentration (pg/mL)	Intra‐day (*n* = 10)			Inter‐day (*n* = 10)		
			Found concentration Mean ± SD (pg/mL)	CV (%)	R (%)	Found concentration Mean ± SD (pg/mL)	CV (%)	R (%)
Lispro	0.999	50	50 ± 1.7	3.5	99.9	50 ± 1.4	2.9	100.0
		125	125.1 ± 1.4	1.1	100.1	126.2 ± 2.5	2.0	100.9
		6000	6021.9 ± 105.3	1.7	100.4	6018.9 ± 76.8	1.3	100.3
		13,000	13,439.1 ± 253.5	1.9	103.4	13,021.5 ± 190.3	1.5	100.2
Human	0.999	50	52 ± 2.3	4.3	104	50.3 ± 0.5	1.0	100.5
		125	121.1 ± 6.9	5.7	96.9	122.7 ± 3.3	2.7	98.1
		6000	6026.4 ± 76.2	1.3	100.4	5982.6 ± 55.9	0.9	99.7
		13,000	13,025.3 ± 192.4	1.5	100.2	12,992.7 ± 90.7	0.7	99.9
Detemir	0.999	50	51.2 ± 1.2	2.4	102.4	51.2 ± 1.0	2.0	102.4
		125	122.2 ± 2.6	2.1	97.8	125 ± 4.1	3.2	100.0
		6000	5991.3 ± 149	2.5	99.9	6060.3 ±115	1.9	101.0
		13,000	13,024.1 ± 225.4	1.7	100.2	13,065.5 ± 155.7	1.2	100.5
Degludec	0.999	50	51 ± 1.5	2.3	102	51 ± 1.5	3.0	102.0
		125	126.1 ± 2.3	1.8	100.9	126.5 ± 2.3	1.8	101.2
		6000	6012.2 ± 111.3	1.9	100.2	6032.4 ± 119	2.0	100.5
		13,000	13,041.3 ± 298.1	2.2	100.3	13,003.2 ± 279.9	2.2	100.0
Glargine	0.999	50	50.6 ± 1.3	2.5	101.2	51.6 ± 1.6	3.1	103.1
		125	126 ± 4.7	3.8	100.8	125.8 ± 3.6	2.9	100.6
		6000	6030.8 ± 168	2.8	100.5	6031.6 ± 53.7	0.9	100.5
		13,000	13,149.1 ± 262.5	2.0	101.1	13,081.5 ± 158.5	1.2	100.6
Glulisine	0.998	50	50.8 ± 1.5	3.0	101.6	50.5 ± 0.9	1.8	101.1
		125	125.4 ± 2.9	2.3	100.3	123.4 ± 1.8	1.4	98.7
		6000	6041.3 ± 65.6	1.1	100.7	6043.6 ± 137.7	2.3	100.7
		13,000	13,155.1 ± 225.6	1.7	101.2	14,861.5 ± 869.8	5.9	114.3
Aspart	0.999	50	51.4 ± 1.0	2.0	102.9	50.3 ± 1.6	3.2	100.5
		125	124.8 ± 1.2	1.0	99.8	124.2 ± 2.1	1.7	99.4
		6000	5958.6 ± 110.6	1.9	99.3	6033.7 ± 58.7	1.0	100.6
		13,000	13,067.1 ± 139	1.1	100.5	13,003.9 ± 162.3	1.2	100.0

#### 3.3.2 Accuracy and Precision

The intra‐day accuracy and intra‐day precision for serum samples were within the ranges of 96.9%–103.4% and 1.0%–5.7%, respectively. The inter‐day accuracy for serum samples was within the range of 98.1%–114.3%, whereas the inter‐day precision was between 0.7% and 5.9% (Table 2). The precision and accuracy met the EMA guidelines [26]. The LLOQ data show that concentrations below the therapeutic reference range can be detected in all cases.

#### 3.3.3 Matrix Effect

The matrix factor was within the acceptable range of ±15% and ranged from 102.7 to 106.9 for lispro, 99.7 to 104.2 for human insulin, 101.7 to 102.4 for detemir, 105.4 to 113.8 for glargine, 100.5 to 114.3 for degludec, 106.1 to 112.3 for glulisine, 105.0 to 107.4 for aspart, and 114.4 for bovine insulin.

#### 3.3.4 Stability

The instability of insulin and its structural analogs in biological matrices, particularly human plasma, is a well‐known phenomenon. Sample stability is an important issue after transporting samples to the laboratory from other hospitals or laboratories. Research suggests that enzymatic degradation, facilitated by enzymes such as IDE and peroxisomal protease, is an important mechanism contributing to this phenomenon [22, 23]. These enzymes, which are found in human plasma, have the ability to rapidly degrade insulin and its analogs. In addition to enzymatic degradation, various chemical factors, such as pH and the presence of metal ions, have been identified to contribute to insulin instability in biological environments. Together, these factors contribute to the rapid degradation of insulin, limiting its biological potency and potentially biasing quantification results, usually due to poor sample storage prior to analysis [23, 33]. In the context of developing analytical methods for insulin analysis, only a handful of authors have addressed stability testing [17, 20, 21, 24, 25, 27, 33–37] a crucial aspect that is often overlooked.

The stability of insulins has been tested in different matrices and under different experimental conditions, with results showing some similarities but also significant differences. Stability in vitreous humor was evaluated in studies by Legg et al. [36] and Beckett et al. [17]. Legg et al. [36] analyzed the stability of six insulins (glargine, lispro, glulisine, aspart, detemir, and human insulin), whereas Beckett et al. focused on glargine, its M1 metabolite, and insulin aspart. Both studies used LC‐MS/MS. The results showed that the stability of insulin in the vitreous humor is highly temperature dependent. Legg et al. [36] found that insulin became undetectable after only 1 day at room temperature, whereas Beckett et al. [17] showed that glargine and its M1 metabolite remained stable when stored at −20°C. These differences highlight the importance of storage temperature and the effect of enzymatic biotransformation on metabolite detection in toxicological analyses.

Stability in plasma has been investigated by several authors, including Xu et al. [37], Baykan et al. [25], Dong et al. [27], Reverter‐Branchat et al. [21], and Abdelwaly et al. [20]. These authors analyzed different types of insulin, such as glargine, degludec, aspart, and other analogs. Xu et al. [37] showed that glargine remained stable at −80°C for 263 days, but its concentration decreased by 15.44% after 25 h and 49 min at room temperature. Baykan et al. [25] emphasized the importance of protease inhibitors and found that K_2_EDTA improved insulin stability at room temperature for up to 24 h. Dong et al. [27] concluded that insulin has high stability at 4°C for 24 h with minimal degradation during freeze‐thaw cycles. Reverter‐Branchat et al. [21] confirmed that degludec is stable when stored at 4°C for 48 h with minimal degradation. Abdelwaly et al. [20] tested the stability of degludec and aspart under various conditions and found that both insulins were highly stable at −20 and −70°C for 30 days.

A comparison of the stability test results shows that low storage temperatures (−20 or −80°C) are critical for maintaining insulin stability in all matrices tested. The results consistently indicate significant degradation of insulins at room temperature within hours to 1 day, as confirmed by studies by Xu et al., Baykan et al., and Legg et al. Studies by Abdelwaly et al. and Reverter‐Branchat et al. demonstrated that degludec has higher stability than glargine, suggesting differences in the behavior of individual insulin analogs under identical conditions [20, 21, 25, 36, 37]. Overall, better stability results were obtained in plasma than in vitreous humor, probably due to differences in matrix composition and the effect of enzymatic degradation.

PIC is used to stabilize plasma proteins and is recommended for proteomic analyses [34]. Other studies have focused on reducing the enzymatic degradation of insulin‐like growth factor 1 using PIC and improving protein stability [24]. Baykan [25] focused on the stability of human insulin at different temperatures and the use of different protease inhibitors. However, the addition of protease inhibitors, such as PIC, did not significantly improve the stability of insulin and, in some cases, led to degradation. In particular, the addition of PIC prior to centrifugation caused hemolysis, which interfered with the measurement of insulin concentration by immunoassay. This confirms that K_2_EDTA is effective in stabilizing insulin at room temperature. Our results indicate that the inclusion of PIC in synthetic plasma has no effect on the degradation of insulins during different testing conditions in this matrix. In addition, the addition of protease inhibitors did not uniformly improve stability under all conditions and, in some cases, had a negative effect on stability. The use of PIC in the stability tests resulted in a reduction in the response of the insulins in MS, leading to a reduction in the sensitivity of the determination by 2D‐LC‐MS/MS.

We chose human plasma for the development of our quantification method because of the higher stability of human insulin in this matrix, particularly at room temperature. Previous research [25] suggested that insulin is most stable at room temperature in plasma collection tubes containing K_2_EDTA.

For quantification, we used a calibration curve that did not include 1% PIC in the artificial plasma. Significant differences in the measured concentrations were observed when using this calibration curve to quantify insulins in artificial plasma containing 1% PIC. These differences were caused by PIC altering the response (peak areas) of both the insulin analytes and the internal standard. Consequently, the presence of PIC significantly reduced the sensitivity of the method. The change in concentration in plasma containing PIC compared to the insulin concentration in artificial plasma alone was lower at time zero and ranged from 17.1% to 100% (data not shown). Therefore, to evaluate the stability of insulins in artificial plasma containing 1% PIC, the measured concentration at time zero was taken as 100% and all subsequent changes were calculated relative to this concentration.

##### 3.3.4.1 Short‐Term Stability

###### 3.3.4.1.1 Short‐Term Stability of Insulins Without PIC Addition

The results of the short‐term stability studies are presented in Table 3. At room temperature, insulin lispro was stable for 24 h at both concentrations, with values ranging from 91.8% to 105.1%. Human insulin was stable for 24 h at 193 pg/mL but decreased to 86.8% after 24 h at 7752 pg/mL. Insulin glargine was stable for 3 h at the highest concentration but fell below the LLOQ at the lower concentration after 24 h. Insulin glulisine was stable for 24 h at 193 pg/mL and for 3 h at 7752 pg/mL, dropping to 86% and 75.1% after 24 h, respectively. Insulin aspart was stable for 24 h at both concentrations. Insulin detemir was unstable at both concentrations after 3 h, with values below the LLOQ and 12.4% after 24 h at 7752 pg/mL. Insulin degludec was stable at both concentrations for 24 h.

**TABLE 3:** Stability of insulins at two concentration levels in artificial plasma with and without 1% protease inhibitor cocktail (PIC) at room temperature at various time points.

Insulin	Concentration (pg/mL)	0 h, change (%)	3 h, change (%)	6 h, change (%)	24 h, change (%)
Lispro	193	96.7	105.1	96.7	100.0
Lispro PIC	193	100.0	84.3	80.0	69.8
Lispro	7752	98.0	95.4	95.5	91.8
Lispro PIC	7752	100.0	99.1	91.0	78.1
Human	193	100.1	102.1	102.4	100.6
Human PIC	193	100.0	86.7	87.7	72.1
Human	7752	99.1	95.4	95.1	86.8
Human PIC	7752	100.0	93.2	86.4	67.4
Glargine	193	99.0	74.2	68.0	<LLOQ
Glargine PIC	193	100.0	51.8	38.8	<LLOQ
Glargine	7752	99.2	83.1	73.4	59.9
Glargine PIC	7752	100.0	86.6	81.4	63.9
Glulisine	193	97.5	102.2	97.5	86.0
Glulisine PIC	193	100.0	78.3	71.1	51.8
Glulisine	7752	96.9	90.4	81.7	75.1
Glulisine PIC	7752	100.0	93.3	76.5	59.6
Aspart	193	100.1	87.3	89.5	93.8
Aspart PIC	193	100.0	85.5	80.3	69.5
Aspart	7752	102.7	100.5	101.6	94.5
Aspart PIC	7752	100.0	95.6	93.1	69.2
Detemir	193	99.5	35.1	<LLOQ	<LLOQ
Detemir PIC	193	<LLOQ	<LLOQ	<LLOQ	<LLOQ
Detemir	7752	100.6	30.2	17.5	12.4
Detemir PIC	7752	100.0	62.8	44.9	60.3
Degludec	193	101.4	105.1	111.6	110.1
Degludec PIC	193	100.0	77.7	65.6	67.1
Degludec	7752	99.2	93.9	88.6	91.8
Degludec PIC	7752	100.0	76.8	49.6	79.3

When refrigerated (4°C), all insulins except glargine, glulisine, and detemir were stable for 24 h. Glargine insulin was stable for 6 h but fell to 46.6% after 24 h at the higher concentration. Glulisine insulin was stable for 6 h at the lower concentration. Detemir insulin was unstable over 24 h, falling below 50%. Degludec insulin was stable for 24 h at both concentrations.

For samples extracted from the autosampler, all insulins except glargine and glulisine were stable at 4°C after 24 h. Glargine insulin was 82% stable at the lower concentration and 92.1% at the higher concentration. Glulisine insulin was stable at 97.6% at the lower concentration but unstable at the higher concentration, with a measured value of 82.2% of the nominal concentration.

###### 3.3.4.1.2 Short‐Term Stability of Insulins With PIC Addition

The results of short‐term stability studies for PIC‐spiked samples are presented in Tables 3 and 4. At room temperature (Table 3), insulin lispro showed slight instability after 3 h, with the concentration decreasing to 84.3% at 193 pg/mL. It showed a significant decrease to 78.1% at 7752 pg/mL and 69.8% at 193 pg/mL after 24 h. Human insulin remained stable at both concentrations for 3 h but decreased to 67.4% at 7752 pg/mL and 72.1% at 193 pg/mL at 24 h. Insulin glargine showed the most significant instability, decreasing to 63.9% at the higher concentration and below the LLOQ at the lower concentration after 24 h. Insulin glulisine was unstable for 3 h at the lower concentration (78.3%). A significant decrease to 59.6% at 7752 pg/mL and 51.8% at 193 pg/mL was observed at 24 h. Insulin aspart was stable at 3 h, but decreased to 69.2% and 69.5% at 7752 pg/mL and 193 pg/mL, respectively, at 24 h. Insulin detemir was unstable at both concentrations, with values below the LLOQ at 193 pg/mL and 60.3% at 24 h at 7752 pg/mL. Insulin degludec was unstable at the lower concentration for 3 h but decreased to 79.3% at 7752 pg/mL and 67.1% at 193 pg/mL at 24 h.

**TABLE 4:** Stability of insulins at two concentration levels in artificial plasma with and without 1% protease inhibitor cocktail (PIC) at 4°C and in the autosampler (AS) at various time points.

Insulin	Concentration (pg/mL)	3 h 4°C, change (%)	6 h 4°C, change (%)	24 h 4°C, change (%)	24 h AS, change (%)
Lispro	193	110.6	112.6	101.8	103.5
Lispro PIC	193	103.7	105.3	87.4	107.1
Lispro	7752	91.8	96.1	91.5	101.4
Lispro PIC	7752	104.7	109.0	94.5	127.4
Human	193	104,4	105.6	108.2	105.1
Human PIC	193	100.4	109.5	87.9	105.9
Human	7752	90.2	98.0	93.2	100.5
Human PIC	7752	97.3	98.6	79.0	123.1
Glargine	193	97.9	93.5	<LLOQ	82.0
Glargine PIC	193	110.8	97.2	<LLOQ	85.9
Glargine	7752	80.2	83.7	46.6	92.1
Glargine PIC	7752	95.9	91.7	69.2	108.0
Glulisine	193	101.6	93.4	76.2	97.6
Glulisine PIC	193	88.8	92.6	57.3	93.2
Glulisine	7752	76.3	80.5	64.5	82.2
Glulisine PIC	7752	93.8	88.8	65.5	101.8
Aspart	193	105.5	98.4	100.4	92.6
Aspart PIC	193	104.5	101.1	81.5	96.3
Aspart	7752	98.8	101.3	94.9	102.0
Aspart PIC	7752	107.7	103.8	89.2	125.7
Detemir	193	54.1	37.8	<LLOQ	98.4
Detemir PIC	193	<LLOQ	<LLOQ	<LLOQ	<LLOQ
Detemir	7752	50.7	33.9	19.7	93.7
Detemir PIC	7752	66.0	53.2	55.1	66.0
Degludec	193	101.8	108.5	98.7	102.9
Degludec PIC	193	88.6	77.2	74.3	91.0
Degludec	7752	92.8	97.7	90.0	98.8
Degludec PIC	7752	85.6	67.5	74.6	99.7

When refrigerated (4°C) (Table 4), insulin lispro was stable for 24 h at both concentrations with values ranging from 87.4% to 94.5%. Human insulin was stable at 4°C for 24 h at the lower concentration. Insulin glargine was stable at both concentrations for 6 h. After 24 h, it decreased to 69.0% at the higher concentration and below the LLOQ at the lower concentration. Insulin glulisine was stable for 6 h at both concentrations, but decreased to 65.5% at the higher concentration and 57.3% at the lower concentration after 24 h. Insulin aspart was stable for 24 h at the higher concentration and decreased to 81.5% at the lower concentration. Insulin detemir was unstable at the lower concentration with a measured concentration below the LLOQ. At the higher concentration, the concentration was below 66% at all time points. Insulin degludec was stable for 6 h at 4°C for both concentrations.

In autosampler stability, insulin glargine, glulisine, and degludec were stable at 4°C for 24 h. Insulin lispro was stable at 107.1% at the lower concentration but showed instability at the higher concentration, which increased to 127.4%. Insulin detemir showed significant instability with concentrations below 66% at both concentrations.

##### 3.3.4.2 Long‐Term Stability

###### 3.3.4.2.1 Long‐Term Stability of Insulins Without PIC Addition

Data from Tables S1 and S2 show that, at −20°C, aspart and degludec were stable for up to 3 weeks at both concentrations, with minimal degradation. At −80°C, insulin lispro was also stable for up to 3 weeks at a concentration of 7752 pg/mL. Insulin glulisine was stable at −20°C for 1 week, but significant degradation was observed after 3 weeks, decreasing from 99.7% to 84.8% at 193 pg/mL. Insulin detemir showed significant degradation when stored at −20 or −80°C.

The addition of 1% PIC to artificial plasma did not contribute to the stabilization of insulins when stored at −20°C or −80°C. This is evident from the data presented in Tables S1 and S2, which show that the stability of insulins under these storage conditions was not improved by PIC.

###### 3.3.4.2.2 Long‐Term Stability of Insulins With PIC Addition

The long‐term stability results of the PIC‐spiked insulins are presented in Tables S1 and S2. Significant changes in the stability of the insulins were observed over 3 weeks when stored at −20°C. Insulin lispro was unstable (80.3%) at the lower concentration (193 pg/mL) after 1 week, and decreased further to 46.3% after 3 weeks. At the higher concentration (7752 pg/mL), the sample was stable at 1 week (112.3%) but decreased to 46.5% at 3 weeks. Human insulin was unstable at the lower concentration from 1 week (70.1%) and decreased to 25.9% at the higher concentration after 3 weeks. Insulin glulisine showed significant instability, with the concentration at the lower concentration dropping below 60% at 1 week (e.g. 51.7%), and at the higher concentration, the values were outside the stable range (31.5% at 3 weeks). Similarly, insulin aspart showed instability, with the lower concentration reaching 39.5% after 3 weeks and the higher concentration falling to 35.8%. The lower concentration of insulin detemir was below the LLOQ, while the higher concentration decreased to 62.2% after 3 weeks. Insulin degludec was unstable at the lower concentration (57.9% at 1 week) and the higher concentration decreased to 51.6% at 3 weeks.

Insulin lispro was stable −80°C at both concentrations throughout the experiment (110.7% at 193 pg/mL and 101.6% at 7752 pg/mL after 3 weeks). Human insulin remained stable at both concentrations with values ranging from 85% to 115% (e.g., 94.1% at 193 pg/mL and 92.5% at 7752 pg/mL after 3 weeks). Insulin glulisine was stable at the higher concentration (95.2% at 3 weeks), but dropped to 79.1% at the lower concentration, indicating instability. Insulin aspart remained stable at both concentrations throughout the experiment, with a minimum value of 93.4%. Insulin detemir showed instability at the lower concentration (<LLOQ), while the higher concentration was outside the stable range (154.5% at 3 weeks). Insulin degludec was unstable at the lower concentration (77.2% after 3 weeks) but remained stable at the higher concentration (125.7% after 3 weeks).

The stability of insulins with added PIC was significantly better when stored at −80°C than at −20°C. At lower temperatures, almost all insulins remained stable, whereas at −20°C, a significant loss of stability was observed after 1 week, especially at lower concentrations.

##### 3.3.4.3 Freeze‐Thaw Stability in Artificial Plasma

###### 3.3.4.3.1 Freeze‐Thaw Stability in Artificial Plasma Without PIC Addition

The results of the freeze‐thaw stability tests are shown in Table 5. Insulin lispro showed good stability at −80°C after freeze‐thaw cycles for up to 72 h, with values of 88.9% and 110.3%. Insulin glargine showed initial stability at 24 h (101.0% at 193 pg/mL and 90.6% at 7752 pg/mL) but significant degradation at 72 h, falling to 94.7% and 87.5%, respectively. Insulin glulisine was stable up to 48 h (99.0% at 193 pg/mL and 92.3% at 7752 pg/mL), but degradation was observed at 72 h, falling to 106.5% and 81.1%, respectively. Insulin aspart showed stability at 24 h (90.4% at 193 pg/mL and 100.0% at 7752 pg/mL) but significant degradation at 72 h, with values of 85.7% and 92.1%, respectively. Insulin detemir showed high instability, with significant increases in concentration at 72 h, reaching 243.7% at 193 pg/mL and 161.5% at 7752 pg/mL.

**TABLE 5:** Freeze‐thaw stability of insulins at two concentration levels in artificial plasma with and without 1% protease inhibitor cocktail (PIC) after storage at −80°C.

Insulin	Concentration (pg/mL)	24 h, change (%)	48 h, change (%)	72 h, change (%)
Lispro	193	109.5	114.0	110.3
Lispro PIC	193	123.2	123.9	96.7
Lispro	7752	96.3	93.7	88.9
Lispro PIC	7752	120.8	121.5	99.2
Human	193	109.3	110.8	108.6
Human PIC	193	105.1	116.6	88.8
Human	7752	100.2	94.6	87.1
Human PIC	7752	111.6	103.5	92.9
Glargine	193	101.0	111.2	94.7
Glargine PIC	193	81.7	76.8	67.4
Glargine	7752	90.6	87.7	87.5
Glargine PIC	7752	108.3	113.9	101.6
Glulisine	193	99.0	107.8	106.5
Glulisine PIC	193	79.0	106.9	92.4
Glulisine	7752	92.3	95.2	81.1
Glulisine PIC	7752	93.1	119.5	95.0
Aspart	193	90.4	98.1	85.7
Aspart PIC	193	111.6	111.6	93.7
Aspart	7752	100.00	101.90	92.10
Aspart PIC	7752	117.4	115.5	99.8
Detemir	193	253.5	349.6	243.7
Detemir PIC	193	115.2	140.6	116.7
Detemir	7752	168.5	195.4	161.5
Detemir PIC	7752	375.0	344.2	285.9
Degludec	193	110.5	108.0	107.9
Degludec PIC	193	122.9	129.8	114.1
Degludec	7752	103.7	101.7	98.6
Degludec PIC	7752	139.3	161.7	140.6

###### 3.3.4.3.2 Freeze‐Thaw Stability in Artificial Plasma With PIC Addition

The stability results of PIC‐spiked insulins after freeze‐thaw cycles at −80°C are shown in Table 5. Stability was assessed on the basis of concentration changes (%) at 24, 48, and 72 h.

Insulin lispro showed slight instability at both concentrations at 24 and 48 h when values exceeded the upper limit of stability (e.g., 123.9% at 193 pg/mL). However, after 72 h, the concentrations decreased to 96.7% (193 pg/mL) and 99.2% (7752 pg/mL), consistent with stability. Human insulin was consistently stable at a higher concentration (7752 pg/mL) with values ranging from 111.6% to 92.9%. At the lower concentration (193 pg/mL), samples were stable at 24 h, but the concentration dropped to 88.8% at 72 h, which is at the lower limit of stability.

Insulin glargine showed significant instability at the lower concentration (193 pg/mL) throughout the experiment, with concentrations dropping to 81.7%, 76.8%, and 67.4%. However, at the higher concentration (7752 pg/mL), samples remained stable with values between 108.3% and 101.6%. Insulin glulisine was unstable at the lower concentration at 24 h (79.0%) but fell into the stable range at 48 and 72 h (106.9% and 92.4%). At the higher concentration, samples were unstable at 48 h.

Insulin aspart was stable at the lower concentration throughout the experiment. At the higher concentration, it was unstable at 24 and 48 h. Detemir showed instability at the lower concentration (193 pg/mL) throughout the experiment. Conversely, at the higher concentration (7752 pg/mL), the values were significantly outside the stable range (375.0% at 24 h and 285.9% at 72 h), indicating significant instability.

Insulin degludec at the lower concentration (193 pg/mL) was only stable at 72 h (114.1%), while the values at 48 and 72 h were above the stable range (122.9% and 129.8%). At the higher concentration (7752 pg/mL), values were above the upper limit of stability throughout the experiment (e.g., 139.3% at 24 h and 140.6% at 72 h).

Overall, the PIC‐supplemented samples showed varying degrees of stability depending on the insulin type and concentration. Stable samples included human insulin at a higher concentration. In contrast, detemir at higher concentrations and degludec was significantly unstable, highlighting that the addition of PIC and freeze/thaw cycles may affect stability depending on the specific insulin.

### 3.4 Analysis of Patient Samples

The validated method was used to determine the concentrations of seven insulins (human, lispro, detemir, degludec, glargine, glulisine, and aspart) in a total of 30 patients. Table 6 summarizes the results of insulin quantification in human plasma samples obtained from the Department of Forensic Medicine, reflecting the use of different insulin combinations commonly administered to patients with diabetes. As patients often use several types of insulin as part of their treatment regimen, the total number of quantifications exceeds the number of samples (*n* = 30). This is evident in the higher number of quantifications for some insulins, indicating the use of combinations, such as basal (e.g., detemir and degludec) and rapid‐acting insulins (e.g., lispro and aspart).

**TABLE 6:** Concentration range of insulins in patient samples.

Insulin	Number of quantifications	Concentration range (pg/mL)
Lispro	4	133.2–6037.3
Human	11	219.1–6065.2
Detemir	11	924.8–4262.8
Degludec	12	999.1–14932.5
Glargine	7	1502.8–5148.7
Glulisine	3	749.5–3419.2
Aspart	7	209.2–4528

**FIGURE 2:**
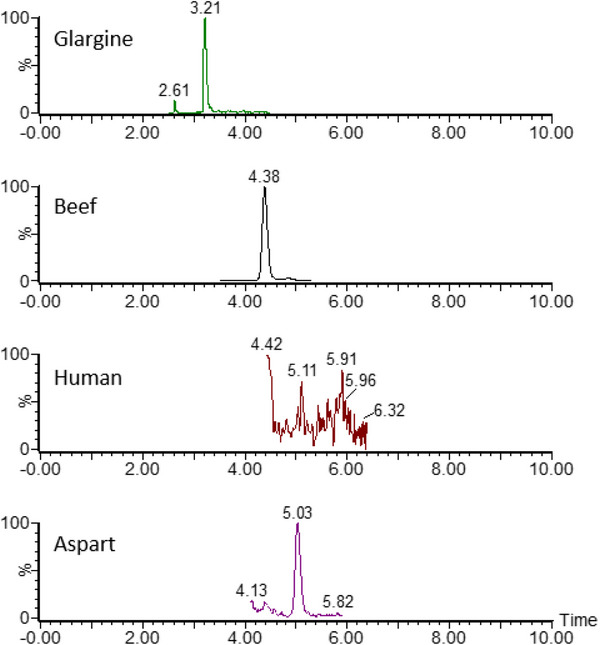
Chromatograms of a real patient sample showing the detection of insulin glargine (2319.5 pg/mL) and insulin aspart (545.8 pg/mL). Human insulin was below the lower limit of quantitation (LLOQ) concentration.

Figure 2 shows the chromatogram of a real patient sample in which insulin aspart and insulin glargine were detected. This combination represents the standard treatment regimen using short‐acting (bolus) insulin aspart and long‐acting (basal) insulin glargine. The combination of aspart and glargine is a proven and effective strategy for glycemic control in patients with diabetes, covering both basal insulin needs and postprandial glycemia, making it the cornerstone of modern insulin therapy [38, 39]. In diabetics on insulin therapy, endogenous human insulin may be absent or undetectable. In patients with type 1 diabetes, the absence of intrinsic insulin is expected due to the destruction of pancreatic beta cells. In type 2 diabetics, endogenous insulin production may be reduced to very low levels or absent in advanced stages of the disease [40].

The measured insulin concentrations covered a wide range. For example, insulin degludec concentrations ranged from 924.8 to 14932.5 pg/mL. The plasma samples were obtained from the Department of Forensic Medicine, where they were collected for analysis in cases of unexplained deaths. This wide range of concentrations highlights the variability of insulin levels and their combinations in different patients, as well as the importance of accurate and sensitive quantification methods. Analytical methods for determining insulin levels in biological fluids vary in sensitivity, which per milliliter can be in the microgram [20], nanogram [11, 41], or picogram range [12], demonstrating the need for highly sensitive techniques to accurately measure these levels.

The forensic context of these samples provides a unique perspective on the variability of insulin levels in different individuals, potentially aiding in the investigation of unexplained deaths.

## 4 Conclusion

The 2D‐LC‐MS/MS method for the determination of human insulin and its analogs (lispro, detemir, degludec, glargine, glulisine, and aspart) is a robust and reliable technique for the quantification of these substances, particularly in forensic cases. The validated approach ensures accurate monitoring and detection, which is critical for both clinical and forensic applications. The simplicity of sample preparation using µSPE, coupled with the efficiency of fast gradient 2D‐LC‐ESI‐MS/MS analysis, allows both single compounds and complex mixtures to be effectively monitored in multi‐method analysis. With MRM and minimal matrix effect, the method achieves the required quantification limits, ensuring high sensitivity and specificity. Although insulin levels are not routinely measured in clinical practice, this method provides an important tool for forensic medicine, aiding in the investigation of unexplained deaths and supporting legal and medical decisions in such cases.

## Supporting information



Supporting Information

## Data Availability

Research data are not shared.
