# Supplementary material for: First Dimension Trap‐and‐Elute Combined with Second Dimension Reversed‐Phase Liquid Chromatography Separation Using a Two‐Dimensional‐Liquid Chromatography‐Tandem Mass Spectrometry System for Sensitive Quantification of Human Insulin and Six Insulin Analogs in Plasma: Improved Chromatographic Resolution and Stability Testing
Source: J Sep Sci. 2025 Feb 10;48(2):e70092. doi: 10.1002/jssc.70092 (PMC11811267; doi:10.1002/jssc.70092)
Supplement: Supplementary file 1 — Supporting Information [file JSSC-48-e70092-s001.docx]

Supplementary Material

Sensitive 2D-LC-MS/MS quantification of human insulin and six insulin analogs in plasma: Improved chromatographic resolution and stability testing.

Pavel Sistik ^a,b,c^, Romana Urinovska * ^a,b^, Klara Handlosova ^d,e^, Petr Handlos ^d,e^, Katerina Andelova ^b,f^, Jan Jurica ^g^, David Stejskal ^b,f^

^a^ Department of Clinical Pharmacology, Institute of Laboratory Medicine, University Hospital Ostrava, 17. listopadu 1790, 708 52 Ostrava, Czech Republic

^b^ Institute of Laboratory Medicine, Faculty of Medicine, University of Ostrava, Syllabova 19, 703 00 Ostrava, Czech Republic

^c^ Department of Clinical Pharmacology, Faculty of Medicine, University of Ostrava, Syllabova 19, 703 00 Ostrava, Czech Republic

^d^ Department of Forensic Medicine, University Hospital Ostrava, 17. listopadu 1790, 708 52 Ostrava, Czech Republic

^e^ Department of Forensic Medicine, Faculty of Medicine, University of Ostrava, 701 03 Ostrava, Czech Republic

^f^ Institute of Laboratory Medicine, University Hospital Ostrava, 17. listopadu 1790, 708 52 Ostrava, Czech Republic

^g^ Department of Pharmacology, Faculty of Medicine and Department of Pharmacology and toxicology, Faculty of Pharmacy, Masaryk University, Kamenice 5, 62500 Brno, Czech Republic

* Corresponding author

E-mail address: romana.urinovska@fno.cz

Romana Uřinovská, PhD., Department of Clinical Pharmacology, Institute of Laboratory Medicine, University Hospital Ostrava, 17. listopadu 1790, 708 52 Ostrava, Czech Republic

Table of Contens:

Figure S1: Structure of human insulin

Figure S2: Structure of lispro insulin

Figure S3: Structure of aspart insulin

Figure S4: Structure of glulisine insulin

Figure S5: Structure of detemir insulin

Figure S6: Structure of glargine insulin

Figure S7: Structure of degludec insulin

Figure S8: ACQUITY UPLC 2D valve in loading mode (0-2 minutes)

Figure S9: ACQUITY UPLC 2D valve in eluting mode (2-8.5 minutes)

Figure S10: Effect of vial type on detector response

Figure S11: Chromatographic separations of insulin lispro and human insulin using 1D chromatography under different gradient elution conditions and with different chromatographic columns.

Figure S12: Chromatographic separation of all insulins at the LLOQ concentration of 50 pg/mL in artificial plasma.

Table S1: Long-term stability of insulins at two concentration levels in artificial plasma with and without 1% PIC at -20°C after 1, 2, and 3 weeks

Table S2: Long-term stability of insulins at two concentration levels in artificial plasma with and without 1% PIC at -80°C after 1, 2, and 3 weeks


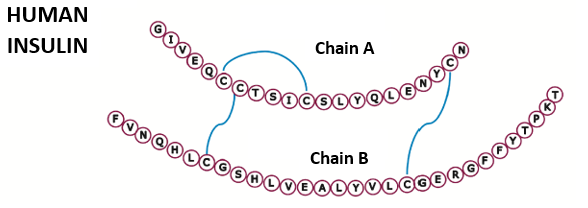


Figure S1: Structure of human insulin


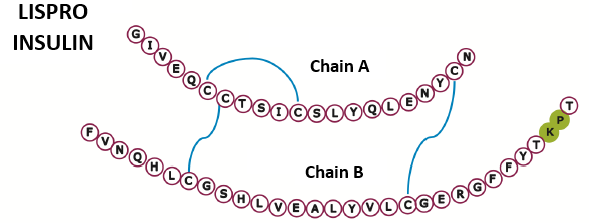


Figure S2: Structure of lispro insulin


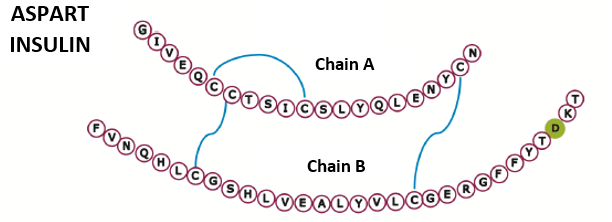


Figure S3: Structure of aspart insulin


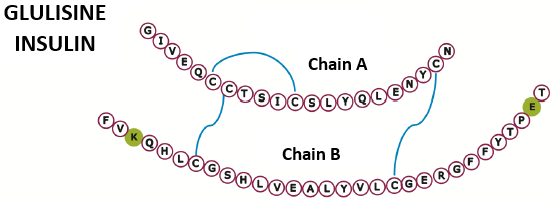


Figure S4: Structure of glulisine insulin


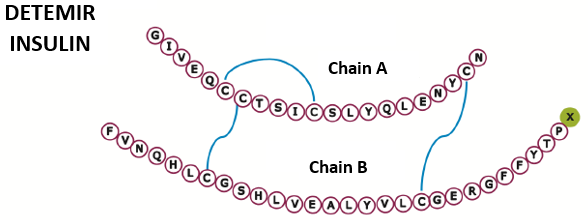


Figure S5: Structure of detemir insulin


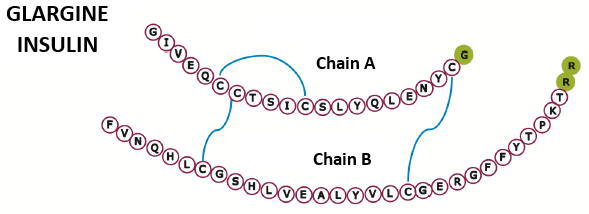


Figure S6: Structure of glargine insulin


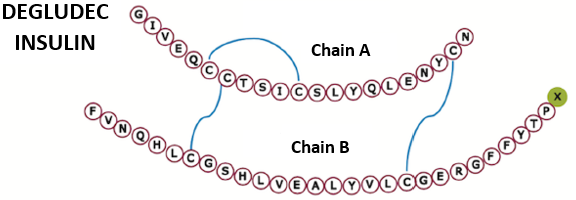


Figure S7: Structure of degludec insulin


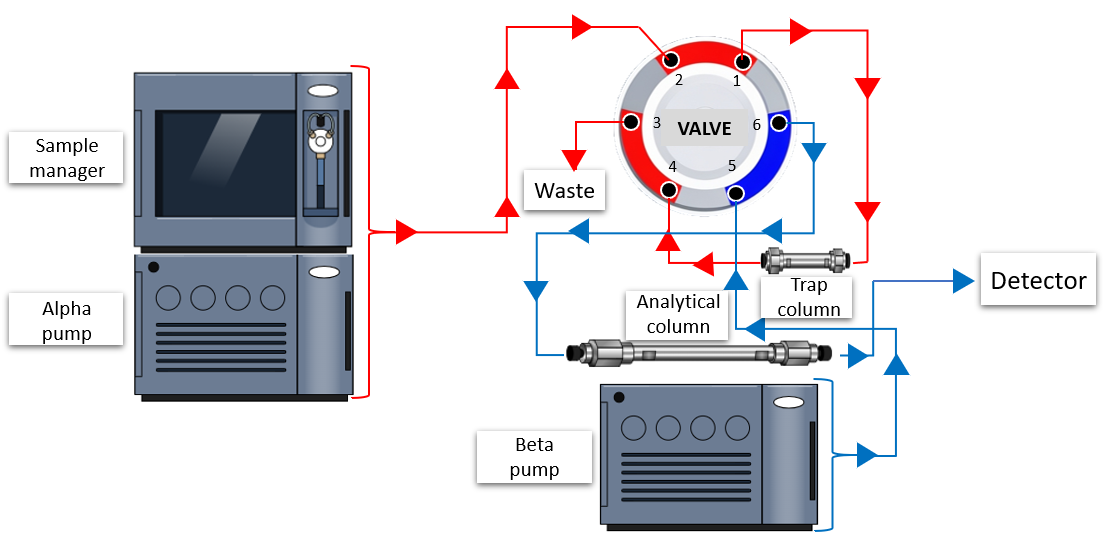


Figure S8: ACQUITY UPLC 2D valve in loading mode (0–2 minutes)


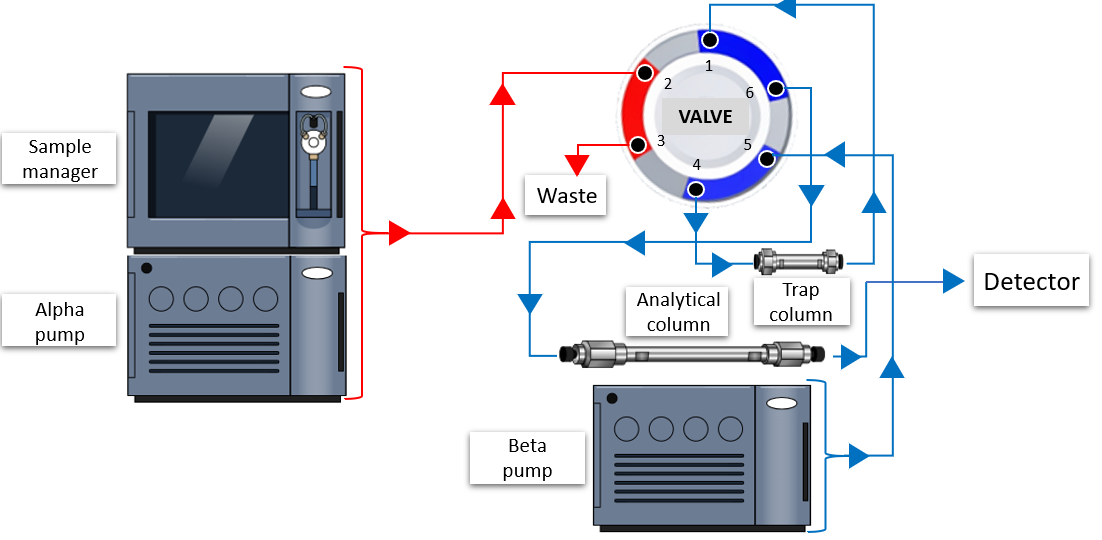


Figure S9: ACQUITY UPLC 2D valve in eluting mode (2–8.5 minutes)


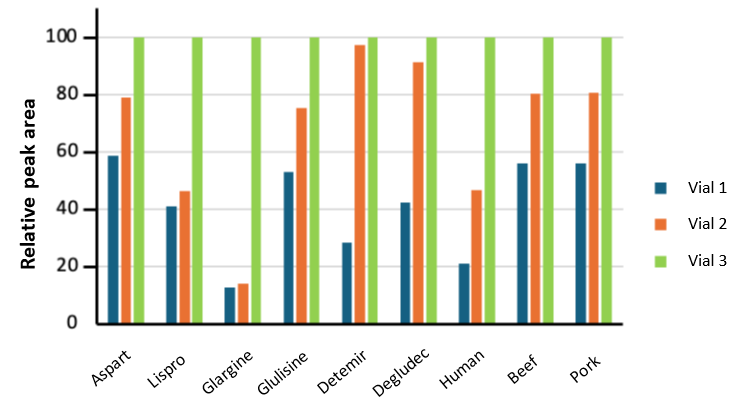


Figure S10: Effect of vial type on detector response


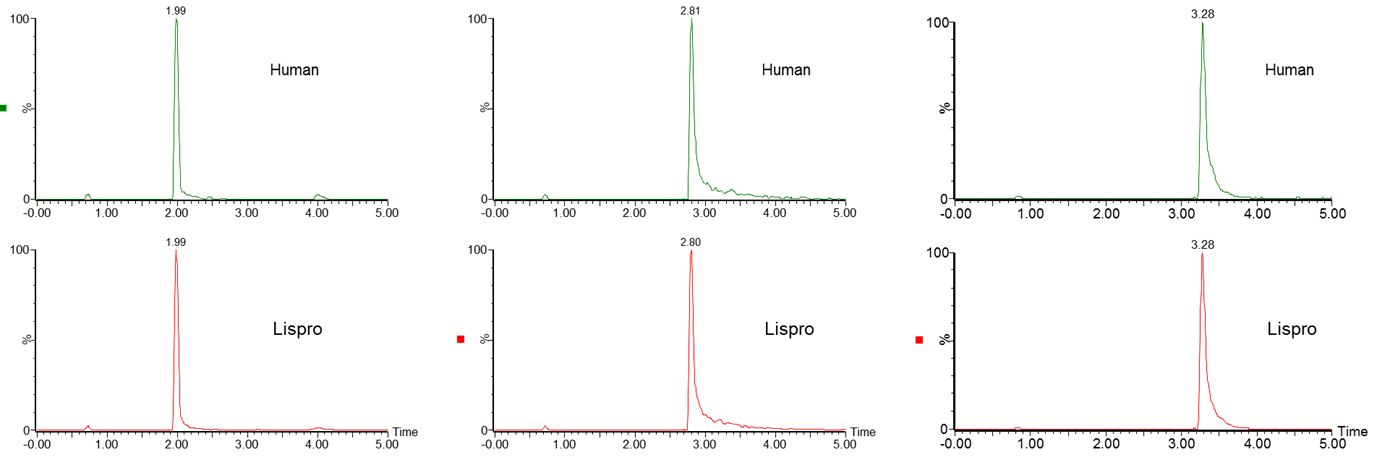


Figure S11: Chromatographic separations of insulin lispro and human insulin using 1D chromatography under different gradient elution conditions and with different chromatographic columns.


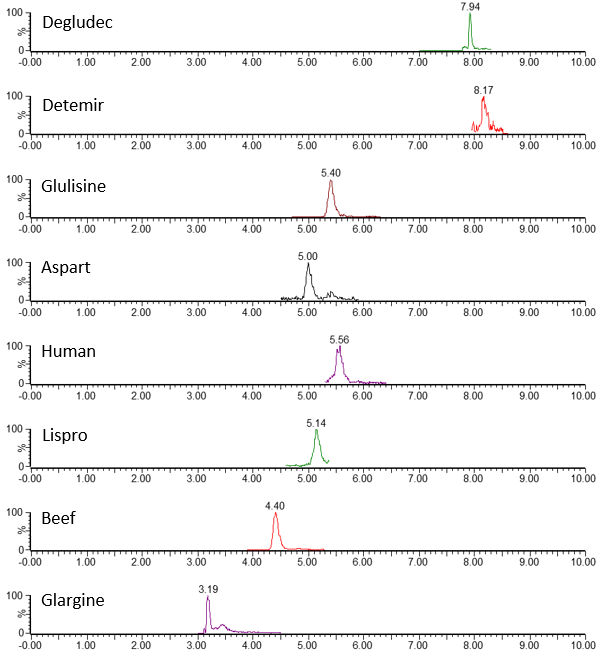


Figure S12: Chromatographic separation of all insulins at the LLOQ concentration of 50 pg/mL in artificial plasma.

**Table S1** Long-term stability of insulins at two concentration levels in artificial plasma with and without 1% PIC at -20°C after 1, 2, and 3 weeks.

| **Insulin** | **Concentration (pg/mL)** | **1 week, change (%)** | **2 weeks, change (%)** | **3 weeks, change (%)** |
| --- | --- | --- | --- | --- |
| Lispro | 193 | 106.8 | 103.9 | 89.3 |
| Lispro PIC | 193 | 80.3 | 68.4 | 46.3 |
| Lispro | 7752 | 92.6 | 85.7 | 79.0 |
| Lispro PIC | 7752 | 112.3 | 81.1 | 46.5 |
| Human | 193 | 105.4 | 106.3 | 102.3 |
| Human PIC | 193 | 70.1 | 59.3 | 47.2 |
| Human | 7752 | 102.0 | 97.8 | 83.8 |
| Human PIC | 7752 | 54.9 | 61.8 | 25.9 |
| Glulisine | 193 | 99.7 | 90.7 | 84.8 |
| Glulisine PIC | 193 | 51.7 | 36.7 | 29.2 |
| Glulisine | 7752 | 86.2 | 84.0 | 79.8 |
| Glulisine PIC | 7752 | 55.6 | 143.0 | 31.5 |
| Aspart | 193 | 105.6 | 107.3 | 101.4 |
| Aspart PIC | 193 | 70.7 | 58.4 | 39.5 |
| Aspart | 7752 | 104.3 | 100.2 | 95.2 |
| Aspart PIC | 7752 | 72.2 | 62.5 | 35.8 |
| Detemir | 193 | <LLOQ | <LLOQ | <LLOQ |
| Detemir PIC | 193 | <LLOQ | <LLOQ | <LLOQ |
| Detemir | 7752 | 27.0 | 24.8 | 21.9 |
| Detemir PIC | 7752 | 71.2 | 71.8 | 62.2 |
| Degludec | 193 | 99.8 | 100.7 | 99.0 |
| Degludec PIC | 193 | 57.9 | 60.2 | 41.2 |
| Degludec | 7752 | 91.2 | 90.7 | 90.2 |
| Degludec PIC | 7752 | 72.3 | 85.6 | 51.6 |

**Table S2** Long-term stability of insulins at two concentration levels in artificial plasma with and without 1% PIC at -80°C after 1, 2, and 3 weeks.

| **Insulin** | **Concentration (pg/mL)** | **1 week, change (%)** | | **2 weeks, change (%)** | | **3 weeks, change (%)** |
| --- | --- | --- | --- | --- | --- | --- |
| Lispro | 193 | 113.3 | 101.0 | | 94.4 | |
| Lispro PIC | 193 | 145.8 | 137.6 | | 110.7 | |
| Lispro | 7752 | 92.4 | 96.9 | | 78.2 | |
| Lispro PIC | 7752 | 112.1 | 111.3 | | 101.6 | |
| Human | 193 | 111.4 | 105.3 | | 102.2 | |
| Human PIC | 193 | 108.9 | 105.8 | | 94.1 | |
| Human | 7752 | 100.1 | 106.0 | | 82.7 | |
| Human PIC | 7752 | 103.9 | 111.9 | | 92.5 | |
| Glulisine | 193 | 103.5 | 102.7 | | 98.8 | |
| Glulisine PIC | 193 | 112.8 | 101.9 | | 79.1 | |
| Glulisine | 7752 | 84.5 | 83.7 | | 75.6 | |
| Glulisine PIC | 7752 | 88.1 | 117.9 | | 95.2 | |
| Aspart | 193 | 106.0 | 103.8 | | 97.8 | |
| Aspart PIC | 193 | 116.5 | 99.6 | | 93.4 | |
| Aspart | 7752 | 104.7 | 97.2 | | 92.8 | |
| Aspart PIC | 7752 | 123.4 | 106.6 | | 108.9 | |
| Detemir | 193 | 64.2 | 50.5 | | 34.1 | |
| Detemir PIC | 193 | <LLOQ | <LLOQ | | <LLOQ | |
| Detemir | 7752 | 63.2 | 54.7 | | 49.4 | |
| Detemir PIC | 7752 | 139.1 | 173.5 | | 154.5 | |
| Degludec | 193 | 101.8 | 97.1 | | 103.9 | |
| Degludec PIC | 193 | 82.3 | 102.2 | | 77.2 | |
| Degludec | 7752 | 101.9 | 95.8 | | 97.5 | |
| Degludec PIC | 7752 | 130.7 | 131.7 | | 125.7 | |
